# Thinking in a Non-native Language: A New Nudge?

**DOI:** 10.3389/fpsyg.2020.549083

**Published:** 2020-09-03

**Authors:** Steven McFarlane, Heather Cipolletti Perez, Christine Weissglass

**Affiliations:** ^1^Department of Philosophy, University of Minnesota at Morris, Morris, MN, United States; ^2^Department of Philosophy and Religion, Broward College, Fort Lauderdale, FL, United States; ^3^Department of Modern Languages and Literatures, Villanova University, Villanova, PA, United States

**Keywords:** foreign-language effect, FLE, bilingualism, decision-making, nudge

## Abstract

The majority of research on learning a non-native language has focused on the personal benefits of being bilingual or multilingual. In this paper, we focus on the potential positive effect of *actively thinking* in a non-native language. Our approach is inspired by recent experimental research suggesting that actively thinking in a non-native language leads to improved reasoning and decision-making, which is known as the foreign-language effect (FLE). We examine the possibility that one could choose to think in a non-native language in order to reap these potential benefits. Integrating this research with research in positive psychology, we explain how doing so might be understood as a type of “nudge,” or intervention that one could use to increase their chances of making autonomous decisions reflecting their own best interest. Nudges have been associated with improved outcomes with respect to many aspects of our lives – for instance sticking to goals, saving money, exercising more frequently, maintaining a healthy diet. It may be that bilinguals can assume an active role in increasing their happiness or well-being by making better decisions through strategic implementation of a non-native language in decision-making contexts. We also discuss the ethics of using the FLE as a nudge when it has beneficial consequences, as there are instances when doing so could be beneficial with respect to public policy as well. For instance, it has been shown that people are less averse to sustainable farming and eating practices (e.g., eating insects) when actively thinking in a non-native language. After reviewing the current research on the FLE, we suggest that further research needs to be done because actively thinking in a non-native language seems to function beneficially in some circumstances but may pose cognitive disadvantages in others.

## Introduction

Research suggests that bilingualism is associated with benefits to cognition and executive functioning. Some of these benefits reflect the fact that both languages are simultaneously active in a bilingual brain (e.g., Marian and Spivey, 2003; Wu and Thierry, 2010). Since bilinguals must constantly employ cognitive control in order to suppress the language they are not currently using and to switch between languages when appropriate, it has been suggested that this leads bilinguals to develop stronger cognitive control than monolinguals (Abutalebi et al., 2008, Abutalebi et al., 2011; Prior and MacWhinney, 2010; Green, 2011; Soveri et al., 2011; Bialystok et al., 2012). Studies have suggested that bilinguals have superior executive functioning (Bialystok et al., 2012) and are better able to switch between tasks (Prior and MacWhinney, 2010) in comparison with monolinguals. Despite the evidence for a bilingual advantage provided by these and other studies, there exists debate in the field as to how robust these findings are. One concern is that experiments showing an advantage for bilinguals are more likely to be published than those that do not (De Bruin et al., 2015; Paap et al., 2015; Lehtonen et al., 2018). Another concern is that sample sizes in these studies tend to be small (Duñabeitia et al., 2014; Paap et al., 2014, 2015). This has led researchers to conduct meta analyses, some of which have confirmed a bilingual advantage (Adesope et al., 2010; Donnelly, 2016; Grundy and Timmer, 2016). While this research is still under scrutiny, being bilingual does seem to provide individuals with at least some cognitive advantages.

More recently, researchers have suggested that *actively thinking* in a non-native language influences the cognitive processes responsible for judgment and decision-making. The idea that we may be able to strategically harness the effect of thinking in a non-native language for our own benefit has already inspired the popular press (Drake, 2012; Greene, 2012; Lieberman, 2017; Watson, 2020). Peñarredonda (2018) writes for *BBC Worklife* that “while at first glance, negotiating in a language other than your mother tongue might seem a disadvantage, it could also make you the most cool-headed person in the room” (2018, section “Potential Issues With Using the FLE as Nudge,” para. 2). Similarly, Skapinker (2018) writes in *Financial Times* that “people working in a foreign language are less susceptible to cognitive bias” and applies this research to the workplace. He states,

an increasing number of people are now working in organizations that operate in English, mixing native and second-language speakers. It is certainly worth thinking about whether people seem more considered, and make more dispassionate decisions, in English than the native speakers do. The non-native speakers may seem less witty, but pay more attention to their opinions (2018, para 15).

The interest in the using one’s non-native language to enhance decision-making is not restricted to researchers, and journalists continue to be inspired to give their readers advice on how best to employ this advantage. Given the widespread interest in using one’s non-native language as a tool to reason better or employing those who can do so, it is important to understand if and how non-native thinking can be harnessed and whether it is always beneficial for us to do so.

In this paper, we discuss actively thinking in a non-native language as a type of nudge toward achieving good outcomes, both for oneself and for greater societal goods, such as sustainable food practices. In this respect, we can understand using this type of nudge in pursuit of good outcomes as a potential element of positive psychology. More specifically, we consider whether actively thinking in a non-native language can serve as a positive psychology intervention (or PPI). We suggest that there needs to be a more nuanced discussion of how it affects reasoning, as research suggests that while it is beneficial in some circumstances, it could pose cognitive disadvantages in others. Finally, we discuss the ethics of implementing active thinking in a non-native language as a nudge to influence the behavior of ourselves and others and conclude that further research is essential for determining the efficacy of the nudge.

## Background

The idea that actively thinking in a non-native language influences the cognitive processes responsible for judgment and decision-making is known as the foreign-language effect (FLE), and it is distinct from the benefits of *being* bilingual generally, as all participants are bilingual, and yet the ones thinking in their non-native language show a marked difference from participants thinking in L1. The original studies on this effect demonstrated that thinking in a non-native language changes how participants respond to reasoning and decision-making scenarios, with the suggestion that it can help to avoid common reasoning errors or decision-making biases (Keysar et al., 2012; Costa et al., 2014a). In this section, we present the foreign-language effect (FLE) in more detail along with some of the proposed explanations for why it exists (i.e., the mechanisms for the FLE). Then, we define “nudges” and provide some common examples of how nudges can be used to improve decision-making^1^. We understand improving decision-making through the lens of improving well-being, and thus connect our discussion of nudges to positive psychology. In section “FLE as Nudge,” we will connect these research areas and discuss how we may be able to harness the FLE to serve as a nudge to benefit ourselves and others. We then discuss some relevant ethical considerations of doing so and present directions for future related research in sections “Potential Issues With Using the FLE as Nudge” and “Directions for Future Research,” respectively.

### Foreign-Language Effect

The FLE was first proposed in Keysar et al. (2012). In their study, bilinguals who were presented with a problem in their native language were more likely to be influenced by the way in which the problem was presented (i.e., the framing effect) than bilinguals who were presented with this problem in the non-native language. In other words, participants who were actively thinking in a non-native language were less sensitive to the framing of the problem independent of the underlying facts. Subsequent research has reported that the FLE reduces other kinds of biases, including the Hot Hand fallacy, where individuals have a tendency to believe that good events will follow other good events (Gao et al., 2015) and illusions of causality, where individuals erroneously conclude that an event caused another event merely because it happened first (Díaz-Lago and Matute, 2018). Actively reasoning in one’s non-native language has also been shown to reduce superstitious beliefs (Hadjichristidis et al., 2019b). Participants who considered events in their non-native language were less likely to attribute negative feelings to “bad luck” events (e.g., breaking a mirror) and less positive feelings toward “good luck” events (e.g., finding a four-leaf clover).

Another domain where the foreign-language effect applies is moral judgment – both in the moral judgment one makes about a case, specifically trolley cases, and with respect to how one judges others’ moral transgressions. In the original moral foreign-language effect research, participants are presented with two moral dilemmas, the trolley case and the footbridge case (Foot, 1978; Thomson, 1986). In the trolley case, a trolley is heading toward five individuals stuck on the track. They will die if the individual does not switch the trolley to an alternative track where only one person will die. Most individuals judge that one should switch the trolley sacrificing the one individual, thereby saving five others. However, people do not tend to make the same judgment with respect to the footbridge case. In this alternate dilemma, one is standing on a footbridge above the tracks. One can save the five individuals on the track below only by pushing a large individual off the footbridge onto the tracks, thereby stopping the trolley. Most individuals do not think that it is morally permissible to push the large man off the footbridge, sacrificing him to save those on the tracks below. In both moral dilemmas, respondents are faced with a choice of whether to sacrifice one individual to save five – where choosing to make this sacrifice is often coded as a utilitarian judgment and abstaining from making the sacrifice is coded as a deontological judgment. However, most people, in their native languages respond differently to the two dilemmas, being much more likely to provide utilitarian responses in the trolley dilemma and deontological responses in the footbridge dilemma (Greene et al., 2001). Researchers have found that individuals are more likely to choose the utilitarian option in the footbridge case in a non-native language than in a native language condition across a wide range of participants from different language groups (c.f. Costa et al., 2014b; Geipel et al., 2015b; Cipolletti et al., 2016), indicating a moral foreign-language effect^2^. Additionally, Geipel et al. (2015a) found that individuals judge moral transgressions and social norm transgressions less harshly in their non-native language than they do in their native language, at least when those transgressions do not involve significant negative consequences (see also Woumans et al., 2020).

Given the research supporting the existence of the FLE, researchers have begun to explore the underlying mechanisms responsible for it, resulting in the following three main hypotheses:

#### The Reduced Emotionality Account

According to the reduced emotionality account, our emotions have a stronger impact on our decision-making processes when we think in our native language as opposed to our non-native language (Keysar et al., 2012; Corey et al., 2017; Hayakawa et al., 2017; Vives et al., 2018; Hadjichristidis et al., 2019a). Since emotions might play into certain heuristics or biased reasoning, reducing emotion by actively thinking in a non-native language would allow for reasoning that is not interrupted or distorted by emotional reactions that one would have experienced reasoning in L1.

#### Metacognition Disruption Account

This account attributes the FLE to a distortion in metacognitive processing (Białek et al., 2019; Muda et al., 2020a, b), wherein there is interference with typical monitoring of first-order cognitive processes. This interference may cause bilinguals who are thinking in their non-native language to engage in more deliberate cognitive processing, rather than the automated, intuitive processing that usually occurs when thinking in one’s native language. According to this view, context is important to determine whether thinking in a non-native language will improve our reasoning. Białek et al. (2019) argues that while this disruption can sometimes result in more rational thinking in cases where our intuitions fail or fall prey to certain cognitive biases, thinking in a non-native language might also impair our thinking in situations where our intuitive processing would typically function appropriately. On the other hand, it may cause us to engage in intuitive reasoning when typically, metacognitive monitoring would intervene and instigate more deliberate cognitive processing.

#### Cognitive Enhancement Account

According to the cognitive enhancement account, thinking in a non-native language engages deliberative cognitive processes, allowing us to avoid common reasoning errors (Costa et al., 2014a; Cipolletti et al., 2016; Corey et al., 2017; Hayakawa et al., 2017; Vives et al., 2018; Jensen Mækelæ and Pfuhl, 2019)^3^. Unlike the metacognition disruption account, this explanation suggests that we can activate deliberative cognitive processes regularly, as if using FLE turns on a switch that improves reasoning or decision-making. Whereas the metacognition disruption account carries the implication that the FLE can be a double-edged sword, the cognitive enhancement account seems to suggest that the FLE will be beneficial to reasoning – or at worst, neutral – in most circumstances.

The uncertainty regarding the underlying mechanism for the FLE is relevant apart from basic research purposes. It may seem that inducing the FLE could only have positive benefits, but if, for instance, the metacognitive disruption account is correct then inducing the FLE might lead to worse outcomes in terms of worse reasoning or judgment. We will return to this question is section “Potential Issues With Using the FLE as Nudge.” For now, we need to define a few other concepts central to our argument – nudges and their relation to positive psychology.

### Nudges and Wellbeing

Having reviewed the FLE and the possibility for improving reasoning and decision-making, we now introduce nudges and tie both the FLE and nudges to positive psychology. Positive psychology is the study of the emotions and actions that contribute the most to human flourishing (Seligman and Csikszentmihalyi, 2000; Linley et al., 2006). In clinical or experimental settings, researchers and clinicians can use PPIs – sometimes a writing or mental exercise, sometimes a behavioral practice – to direct attention in positive ways or to establish positive habits (Seligman et al., 2005; Sin and Lyubomirsky, 2009). The goal of PPIs in a research context is to determine what sorts of interventions influence our well-being, and the goal of PPIs outside of a research context is to help people flourish. Our aim is to expand upon the idea that the FLE might be used as a nudge toward positive outcomes, or a type of PPI, and thus complements research in positive psychology.

Nudges are features of a decision-making context that influence how people make decisions. Importantly, while altering an individual’s behavior, nudges “do not forbid other options or significantly change incentives” (Thaler and Sunstein, 2008, p. 6). To count as successful nudge, the interventions must be easy to employ (e.g., low cost, requiring little effort). Understanding nudges and knowing how best to employ them is considered in society’s best interest because they can help individuals alter their behavior to act in ways that are in their own best interest or in the interest of the public good (e.g., public health) without undermining their own autonomy – their ability to choose to act in accordance with them or not^4^.

Nudges come in many forms and are used for many purposes (Lunn, 2014; Sunstein, 2014). Common nudges include using defaults (e.g., needing to opt out of being an organ donor, rather than needing to opt in), simplifying complex forms or procedures (e.g., tax forms), arranging environments so as to draw attention to choice-worthy items (e.g., stocking attractive items at eye height at the grocery store while putting cheaper generic brands on shelves close to the floor), and adopting useful heuristics (e.g., always round up to the nearest dollar while shopping). Nudges in themselves belong to a general category of non-coercive influence on the psychology of choice, and it is possible for nudges to have good, neutral, or bad outcomes. In this section, we discuss the possibility of using nudges for good, and in section “Potential Issues With Using the FLE as Nudge” we turn to the question of harmful nudges.

It is possible for nudges to directly contribute to well-being, as there are nudges with explicit connections to positive psychology research. For instance, Seligman et al. (2009) explore the power of PPIs in an educational context, including the effects of interventions involving mental and writing exercises, such as writing down three good things that happened each day (p. 301). A prompt to write down three good things per day is plausibly both a PPI and a nudge, as it is a non-coercive influence that could increase flourishing. Likewise, nudges can be used to establish healthy habits, such as a mindfulness smartphone app (Howells et al., 2016)^5^. Not every nudge is a PPI, and not every PPI is a nudge. Some PPIs could potentially be coercive, such as mandatory positive psychology training or state-mandated medications for people with severe mental health problems. These are examples of PPIs, as presumably these activities would improve individuals’ well-being, but they are not nudges, as nudges must, by definition, be non-coercive. Likewise, some nudges are not PPIs, since they are not intended to enhance flourishing (e.g., requiring gym members to cancel their membership in person) But there is an intersection between PPIs and nudges worth exploring.

One more distinction will help in the discussion ahead. The distinction relates to what sort of thing is being acted upon as an independent variable – something in the external environment, or something internal to a person’s psychology directly? Nudges that intervene on environmental stimuli are what we might call *external* nudges, and nudges that involve interventions that directly target our cognitive framing or processing of environmental stimuli, are *internal* nudges^6^. External nudges affect primarily non-mental or environmental elements of choice architecture. Examples of external nudges include using a warning label, listing nutritional information, or sending text reminders. In the end, all nudges have an influence on internal states of our psychology. But in the case of external nudges, one enacts changes to internal cognitive states *via* interventions on things outside of our heads. Internal nudges affect primarily mental or cognitive elements of choice architecture directly, rather than through external elements. For instance, someone who is feeling down might look at a photograph taken during their honeymoon and get a dose of happy nostalgia as the memories come flooding back. Or a person in the same initial sad state can simply intentionally remember the honeymoon without looking at any photos, knowing that this always puts them in a better mood. The former would count as an external nudge, as the internal change happens via looking at the photo, and the latter an internal nudge because the nudge is simply an intentional activation of memory without the use of environmental stimulus.

In the following section, we will explain how inducing the FLE can be understood as an internal nudge to improve our own decision-making and, perhaps, be instituted to advance the public good (e.g., through sustainable food practices). As institutions and society-wide flourishing fit within the positive psychology framework, it is important consider how the FLE can relate to flourishing at both the individual and societal levels.

## FLE as Nudge

Given the research suggesting that actively thinking in a non-native language leads to better decisions by helping to avoid common biases, it stands to reason that bilinguals could benefit from choosing to make decisions in their non-native language. In this section, we explore some possible situations in which intentionally engaging the FLE could be considered a “nudge” toward adopting behaviors that encourage positive outcomes or lead to a decision that reflects their best interest. Additionally, we will discuss how the FLE can be used to benefit society as a whole, as researchers have suggested that we might use the FLE for purposes related to public or environment policy (U.N., banks, or sustainable eating practices as found in Geipel et al., 2018).

Consider another example from Keysar et al. (2012). In addition to diminishing the framing effect (see section “Background”), they found that actively thinking in a non-native language reduced the degree to which bilinguals allowed their fear of losing something (i.e., loss aversion) to unduly influence their decisions. Specifically, bilinguals thinking in their non-native language were more likely to accept favorable bets than bilinguals thinking in their native language. If actively thinking in a non-native language can reduce loss aversion, bilinguals may want to intentionally engage the FLE to improve their chances of making a decision that reflects their best interest (i.e., “nudge” themselves toward a better outcome). For example, suppose Vinny can make an investment with a 50/50 chance to get 250% return on his investment and a 50/50 chance to get zero return (and that this is money that Vinny can comfortably lose). According to some normative models of decision-making of how one *ought* to choose, such as expected utility theory, Vinny ought to make this investment^7^. Suppose that were Vinny to consider the investment opportunity, the psychological reality is that he would be loss averse and choose to let it go by. Could he intentionally engage the FLE to reduce loss aversion and choose to make the investment?

Additionally, the benefits of using the FLE as a nudge might be able to be extended to society as a whole. For example, Geipel et al. (2018) found that bilinguals who were asked about sustainable food practices (e.g., eating insects or drinking recycled water) in their native language were more opposed to engaging in these practices than those who were asked about them in their non-native language. The authors propose that:

The main barrier to the adoption of these products is the disgust they elicit. Although recycled water is technically clean, in people’s minds it is dirty because it was once in contact with a disgusting entity. Similarly, surveys on artificial meat and insect-based food link refusal to adopt these products to feelings of disgust (Geipel et al., 2018, p. 3).

Given that sustainability is an important and relevant issue, the fact that participants were more open to it when thinking in their non-native language lends us to say that it may be beneficial to society if some of these decisions were made in non-native language (Bonini et al., 2018, p. 819–820).

The promise of using FLE as nudge for increasing personal well-being or for greater societal goods is apparent. To see more concretely how the FLE might be used, recall the distinction between internal and external nudges. A potential use of the FLE as an internal nudge. might take the form of enacting an internal rule: *When making an important decision, consider it using a non-native language*. Or suppose Vinny knows he has a history of regretting our decisions when it comes to specific domains (say saving vs. spending money). Vinny might adopt a heuristic before making a purchase *consider in a non-native language whether it would be better to buy this item or to save*. Or perhaps friends and colleagues can play a role in our decision-making procedure as external nudges. For example, one could ask one’s partner to present in a non-native language the question “should we bid for this house or continue to rent?” “Deberíamos hacer una oferta por esta casa o seguir alquilando?” (this option would add an external nudge component). We might choose to read important documents in a non-native language or conduct business meetings in shared non-native languages, as well. Thinking in a non-native language directly, absent environmental stimulus can be an internal nudge, and reading a text in a non-native language could be an external nudge that activates thinking in that language.

To summarize, it may be possible to use the FLE to help us make better decisions toward our own well-being. Thaler and Sunstein famously claim that nudges can helps us make better decisions regarding “health, wealth, and happiness,” and to the extent that the FLE can be used as such a nudge, it shares in this potential. If this is right, then the FLE as a nudge nicely complements the aims of positive psychology – there is a shared interest in pursuit of encouraging positive episodes, traits habits, and institutions. We encourage more research at the intersection of the FLE and nudges to see where positives opportunities lie.

Though there is promise, it is important not to fall prey to excessive optimism. In the next section, we examine some limitations with respect to our current knowledge of the FLE and provide reason to be more cautious about overly simple recommendations to use the FLE as nudge. Specifically, it is important to consider how attempts to nudge can backfire, causing negative outcomes or instilling bad habits or traits that are antithetical to human well-being.

## Potential Issues With Using the FLE as Nudge

Our discussion for the possibility of misuse of FLE as nudge has two parts. First, we will address some problems that one could encounter when attempting to use the FLE to improve their own decision making, which would be considered a *self-directed nudge*. Then, we will discuss some issues that could emerge when someone tries to use the FLE to improve someone else’s decision making, which would be considered an *other-directed nudge*^8^.

### Potential Issues With the FLE as a Self-Directed Nudge

Our primary concern with using the FLE as a self-directed nudge is that, given our current state of understanding, we might possibly instigate as many “sludges” as nudges. Sludges are features of decision-making contexts that lead to worse outcomes (Thaler, 2018; Sunstein, 2019). Making tax filing directions overly difficult, confusing street or parking signs, or requiring many difficult steps to opt out of a subscription qualify as sludges. Sludges are more likely to induce negative emotions, such as frustration or anxiety, and to interfere with forming positive habits or engaging with healthy behaviors.

There is some evidence that thinking in a non-native language can *negatively* affect one’s judgment. For example, Białek et al. (2019) found that bilinguals who completed logical reasoning tasks in their non-native language were less accurate than those who did so in their native language. The authors explain that thinking in a non-native language may inhibit one’s ability to recognize when the validity of an argument needs to be evaluated. This research suggests that bilinguals should not use the FLE as a self-nudge in logical reasoning tasks and/or situations in which the validity of an argument is not guaranteed.

Even when the FLE is not actively harmful, suggesting that the FLE is always an effective nudge may itself act as a sludge. Some research suggests that the thinking in a non-native language has *no effect* on critical thinking tasks (e.g., the cognitive reflection task) or tasks involving representative bias or the conjunction fallacy (Vives et al., 2018). Given this research, there are two possible outcomes of using the FLE as a self-nudge in these tasks. At best, the outcome would be neutral (i.e., their decision would not be better or worse than it would have been had they made the decision in the native language). However, they could also too readily accept their decision simply because they used their non-native language, having decided incorrectly that using their non-native language would always result in a more rational decision. If their decision would have been different had they used their native language, there would exist the possibility that they made the wrong decision.

Advocates for using the FLE in the popular press may have presumed that the Cognitive Enhancement account is correct – that using the FLE simply improves reasoning or decision-making. However, this assumption is too hasty. It may be that the FLE is impairs reasoning or has no discernable effect. It is important to know when and why the FLE occurs before making sweeping recommendations for widespread use.

### Potential Issues With the FLE as an Other-Directed Nudge

In this subsection, we look at two sorts of concerns with the ethics of using FLE as nudge. First, we look at whether we can be sure that using the FLE *improves* moral judgments by making them more rational, and then we turn to the ethics of nudging others.

In pursuit of beneficial nudges, we must be wary of the possibility that activating the FLE might at times act as a sludge. Theoretical research can find its way into the popular press and opinion, and it is important to make sure that limitations on what researchers know place constraints on what might turn into popular advice, and researchers must play a part in preventing potentially reckless use of this research. This could happen in a few ways. Take the reduced emotionality account of the FLE first. This account says that using the FLE attenuates the salience of moral and socio-cultural norms. If so, nudging yourself with the FLE could lead to undesirable outcomes. For example, if one thinks that there are relevant moral differences between the trolley and footbridge moral dilemmas such that one ought not maximize lives saved in the footbridge case even if one ought to do so in the trolley case, and that considering these cases in a non-native language reduces our ability to detect those differences, then one might conclude that we should not engage in moral reasoning in a non-native language. And, indeed, some popular news sources have concluded this – a writer for an online blog, The Language Nerds (2020), stated that you are “more likely to make immoral decisions while speaking a second language” and that “the languages we speak interfere with and direct our moral choices”. While one might argue that utilitarian moral judgments are not *immoral*, it is still clear that at least the writer of this blog would consider the FLE to be a sludge rather than a nudge with respect to moral deliberation. In contrast, Lieberman (2017) from *Travel and Leisure* states doing so makes you more “a more logical, utilitarian reasoner” and that actively considering these scenarios in a non-native language makes us “able to more clearly consider the consequences of our decisions, and be our most rational selves.” The fact that popular news sources are drawing general conclusions on the moral implications of using a non-native language, and providing contradictory advice regarding the morality of thinking in a non-native vs. native language on the basis of FLE research illustrates the importance of acknowledging how little researchers currently know about the FLE and the mechanisms that underlie it. Therefore, some caution is in order to provide time to better understand when and how the FLE might be used as a positive nudge rather than a sludge.

We turn now to the ethics of nudging others. There are difficult ethical questions regarding the relationship between mere influence on the one hand and manipulation on the other. Of particular interest here is whether those being nudged are aware that they are being nudged and whether they are willing to be nudged. Furthermore, we can divide cases into those that are paternalistic, where we are nudging others for their own good vs. cases where we are nudging others for the sake of the greater good, irrespective of the individual’s good.

To demonstrate the relevance of awareness and consent in evaluating paternalistic other-directed nudges, let’s imagine that a man has been accused of a crime – call him Defendant – and he is going to have to make several important decisions in the next few weeks. He is a native speaker of German and a non-native speaker of English. He claims to feel more comfortable and confident communicating in German. In preparation for the trial, he meets with a lawyer who is equally comfortable and confident communicating in both languages. The lawyer knows about her client’s linguistic situation before their first meeting, and she also knows about the research showing that actively thinking in a non-native language reduces the effect of some common reasoning errors^9^. Based on this information, she wants to use English when she communicates with her client in the hopes that he will be more likely to make decisions that are in his best interest. Let’s look at a few different scenarios to see how awareness of a nudge and willingness to be nudged can interact to create situations that differ in terms of how ethical they are.

We can imagine a matrix of Defendant’s condition with respect to his understanding and consent to the effects of actively thinking in a non-native language. In other words, we can categorize the Defendant as being aware or unaware of the FLE and either consenting or not consenting to the influence the FLE will have on his thinking. For the sake of concision, we illustrate with a description of a few scenarios, though more can be constructed.

#### Scenario 1: Aware and Voluntary

The lawyer asks Defendant, who happens to be very familiar with the research on the FLE, which language he would prefer to use in the meeting, and he chooses English. They conduct the rest of the meeting in English. In this case, he is *aware* that making these decisions in his non-native language is beneficial, and his participation is *voluntary*, since he chose to conduct the meeting in English.

#### Scenario 2: Aware and Involuntary

The lawyer conducts the meeting in English. Defendant happens to be very familiar with the research on the FLE, but he asks her to switch to German because he feels more comfortable speaking in German. She says “no” because she wants him to make decisions in his best interest. They conduct the rest of the meeting in English. In this case, he is *aware* that making these decisions in his non-native language is beneficial, and his participation is *involuntary*, since he did not want to continue the meeting in English.

#### Scenario 3: Unaware and Involuntary

The lawyer begins the meeting in English, but Defendant asks her to switch to German because he feels more comfortable speaking in German. She says “no” because she wants him to make decisions in his best interest. They conduct the rest of the meeting in English. In this case, he is *unaware* that making these decisions in his non-native language is beneficial, and his participation is *involuntary*, since he did not want to continue the meeting in English.

The three scenarios above show the different ethical dimensions that can arise when using the FLE as a nudge. If Defendant is aware of FLE and agrees to it (Scenario 1), there is little ethical concern. But if Defendant is aware and would not prefer to undergo the effects of non-native thinking (Scenario 2), if there are such, then it is far from clear that his attorney should supersede that preference, even if she thinks it is for his own good. Scenario 3, where Defendant is unaware of the effect, but would not agree to it if he knew, also raises red flags. Surely, Defendant’s wishes with respect to how he wants to reason and make decisions carries ethical weight.

These issues escalate when we turn to other-directed nudges for the greater social good. Using the FLE is thought to cause changes in how people think, and while there are ways to change others’ thinking that are permissible or even laudatory (i.e., providing education), ethically dubious methods lurk as well. In particular, one might be concerned about manipulating others via strategic application of the FLE, especially when the speakers who will be affected are unaware of the influence of the FLE.

To focus discussion, let us use Geipel et al. (2018)’s suggestion that the FLE causes people who are aware of the features of these products to be less averse to sustainable food practices, such as eating insects or drinking recycled water. Suppose that someone is aware of the relevant properties of these products and is averse to ingesting them when presented the choice in her L1 but is not averse if the choice is presented in a non-native language. Is this a case of unethical manipulation?^10^ If so, then surely it matters in the quest for both healthy and flourishing individuals as well as larger institutions.

On the one hand, it may seem that the initial aversion is due to an irrational, emotion-based bias. Using the FLE as a nudge might dampen this irrational bias, causing us to be more responsive to the positive, sustainable features of adopting these food practices. (This is the reduced emotionality account at work. See Geipel et al., 2018, p. 6–7). However, there is a danger in characterizing others’ positions as biased and emotion-based, and therefore justifiably influenced via nudges. Supposing that people had full information and were averse before the nudge intervention, we may wonder if reducing their emotional reaction is a justified form of influence. Or perhaps the metacognition disruption account is correct. Then using a non-native language may cause people to be less averse due to our metacognitive monitors overlooking what would be a first-order intuition conflict when choosing to enact the sustainable food practices. Interference with metacognitive monitoring has the potential for objectionable manipulation. The details behind the causal mechanism responsible for the FLE and how attempted nudges interact via that mechanism matter.

We submit that the following are important ethical considerations when one is attempting to nudge others via the FLE:

1. Transparency and prior consent to using a non-native language to influence thinking in environments outside of the laboratory, where these decisions might have real stakes, is essential.
2. Understanding the causal mechanisms and how FLE nudges interact with them is important.

Before committing to using the FLE to nudge people to engage in more socially cooperative or greener behavior, it is important to think carefully about how the FLE works and how it is being used. If the FLE simply makes someone more rational – perhaps cognitively enhanced or less susceptible to irrational emotions – by their own lights, then it may seem that there is no ethical issue or moral trade-off. It may seem like a positive sum intervention. However, there is the potential for paternalism or problematic manipulation if others determine which of our reactions or emotions are irrational or due to inappropriate emotional biases. If people have a disgust reaction which causes them to be resistant to consuming recycled water, then there is an implicit value judgment to claim that this disgust reaction is irrational or that it ought to be countered via the FLE, as if it were a problem to be solved. It may be the correct value judgment, but it is important to recognize the ethical trade-off.

The matter is even more ethically fraught if the FLE has a potential to work as a sludge. If we are making people less rational, perhaps by inhibiting their metacognitive abilities to monitor their own intuitions appropriately, then influencing others via the FLE might qualify as manipulating others, in part by diminishing their abilities rationally make autonomous decisions, for the sake of the ends of the ones doing the influencing. There are ethical arguments to be had about when, if ever, bypassing others’ autonomy is appropriate for the greater good (Savulescu, 2007). Similarly, one can debate the benefits of PPIs and weigh them against bypassing autonomy, but this must be done explicitly and with a full hearing from those who doubt that PPIs outweigh potential costs. At the very least, the exuberant recommendations to think in a non-native language to be rational or a rush to institute widespread green nudging deserves more scrutiny and ethical consideration.

## Directions for Future Research

The question of where FLE might work best as a nudge toward positive outcomes is difficult to judge, as it depends on when nudges in general are effective, when the FLE is effective, and what happens when nudges and the FLE intersect. In this section, we will discuss how different lines of research could help to address whether (and how) the FLE can be used as a nudge.

The first consideration is that, as mentioned in previous sections, researchers do not yet have a full grasp of when the FLE appears and when it does not. Actively thinking in one’s non-native language affects loss aversion but appears not to influence people’s thinking with respect to the conjunction fallacy. And we do not know exactly why, with no mechanism for the FLE sufficiently confirmed at the moment. Further research needs to be done to understand the mechanism underlying this effect.

Another consideration for future research relates to language proficiency. Keysar et al. commonly assert that the FLE works most effectively, or even perhaps exclusively works, for unbalanced bilinguals – those who learned their non-native language in a different context than they acquired their native language (c.f. Hadjichristidis et al., 2019a). The implication is that native-like mastery of a non-native language (e.g., as in the case of balanced bilinguals) may not be conducive to the FLE. Proficiency could also play a role when native-like mastery has not been achieved. For example, beginning language learners with low proficiency may not fully comprehend cases presented in their non-native language. So, clearly, a language learner must have a certain level of proficiency in order to reason effectively in their non-native language. However, while a larger role for proficiency (or context of learning) has theoretical merit, up until this point we are unaware of any research confirming this. If it is true, for example, that the context of learning is important to whether the FLE works or not, then we may have a better understanding of the mechanism underlying the effect and a better understanding of how it could be used as a nudge. This could even inform language pedagogy, as it may be advisable for individuals to learn another language later in life.

Additionally, we do not have a firm grasp of some other temporal properties of FLE interventions. We do not yet have enough information regarding how long after beginning to use a non-native language it takes for the FLE to kick in or how long the effects last afterward. Another question to consider is whether there a minimum amount of time one must think in a non-native language to produce the FLE. The temporal aspects of the FLE is an area where more research would be valuable.

A separate question relates to perceiver effects. If you know that you are using the FLE in order to nudge yourself, will it still work? Most research on the FLE does not consider a speaker’s awareness of the effects of the FLE. Perhaps knowing that one is attempting to be more rational via the FLE will cause one to believe one is more rational, even when one isn’t. Or perhaps believing that one is being more rational will cause the FLE to be a placebo-like effect, causing a change in speakers’ choices, but without the FLE itself being responsible for those changes. At this point, this matter remains unclear, and, if we want to use the FLE as a self-directed nudge for our own self-interest, it must be efficacious when we have knowledge of the effect.

The FLE as nudge is probably most likely to be effective when a slight shift in perspective while looking at the same picture changes the choice outcome. After all, it would be very strange if you developed a radical change in your preferences and values just by switching to a non-native language. A common feature of many scenarios where the FLE is found is that the relevant properties of both choices are apparent from the start. For instance, the odds in the coin-tossing loss aversion experiment, the numbers of who will be saved and who will die in the framing experiments and moral dilemmas, more. In short, the FLE is commonly found in dilemmas where the consequences are clear, beforehand, and multiple options have some sort of appeal to them. All participants are already split, to some extent. But relevant properties become just a bit more salient, enough to sway the overall decision. In hard cases, or in cases where it easy to miss important features, it is not clear what purchase the FLE might have.

## Conclusion

We have examined the prospects of using the foreign-language effect as a nudge. The evidence so far is mixed, but there may be the potential to use the effects of actively thinking in a non-native language for our own good. Future research should investigate the exact mechanism through the FLE works. If the metacognition disruption account is correct, and the FLE distorts our ability to recognize conflicts in intuitions, then using the FLE could be as likely to act as a “sludge” as it is to work as a nudge. If the reduced emotion account is correct, then we ought to be careful to use the FLE as a nudge only in cases where increased emotion is harmful, and it is very challenging to systematically predict when these cases occur. Further research is also needed to determine the limits on the FLE’s effective capacity to change how we reason and for how long.

Positive psychology has identified several factors that contribute to an individual’s happiness, well-being and/or positivity. Some of these factors include establishing and maintaining healthy relationships and exercising. In addition, engaging in appropriately challenging tasks that require a higher level of skill has been shown to positively impact one’s level of personal satisfaction. Similarly, learning and using a non-native language may have additional benefits beyond its intrinsic value. If the FLE can be used as a nudge, then learning a non-native language can help us lead happier, more successful lives.

## Author Contributions

SM had the original idea for this article. All authors contributed equally to the research for and writing of this article. All authors contributed to the article and approved the submitted version.

## Conflict of Interest

The authors declare that the research was conducted in the absence of any commercial or financial relationships that could be construed as a potential conflict of interest.

## Footnotes

1 Researchers working on the FLE and nudges have separately tied their research to Dual-Process models of reasoning. Some interlocutors focus on aspects of Dual-Process models as well (Saghai, 2013; Osman, 2016). Perhaps an understanding of Dual-Process models enriches understanding of these concepts, but we think the main ideas we want to discuss can be covered without layering a third construct in between nudges and the FLE for the purposes of this paper.

2 Recent studies on moral judgment investigating the role of language mode have shown that bilinguals using their native language exhibit similar response patterns to participants in older studies not investigating this variable (e.g., Greene et al., 2001). This suggests that the participants from the older studies were likely reasoning in their native language, although we cannot be certain of this.

3 While the CEA is addressed in the research cited here, it is important to note that the authors do not necessarily argue in favor of this account.

4 Autonomy and autonomy supporting behaviors have been tied to increased well-being and autonomy as a trait arguably belongs in the canon of positive psychology traits (Chirkov et al., 2011).

5 For a review of research involving apps nudging users to perform positive psychology exercises, see Bakker et al. (2016).

6 Grüne-Yanoff and Hertwig (2016) call internal nudges that enhance reasoning or decision-making “boosts.” For our purposes, we are treating boosts as subspecies of the larger phenomenon of nudges that primarily work via effects on internal cognitive processes.

7 See Jeffrey (1983) for a classic statement. We do not endorse any particular theory of rational decision-making, and only use expected utility as a mainstream model for purposes of illustration.

8 One might wonder how the internal vs. external nudge distinction maps on to the self-directed vs. other-directed nudge distinction. One can attempt to influence one’s own choices with internal nudges (intentionally counting in one’s head to ten so as not to make a decision in anger) and external nudges (looking at a photo from one’s honeymoon to cheer up). One can externally nudge others in lots of ways (for instance, the Surgeon General attempts to nudge people not to smoke by putting large warnings of health hazards on cigarette packaging). It is unclear if it is possible to internally nudge others, though it is common to think that prayer or positive thoughts have external impacts on the world, potentially including influencing other peoples’ decisions.

9 We are assuming for the sake of argument that the FLE really does reduce the effect of common reasoning errors, as it makes the strongest case for nudging others for paternalistic reasons. If it is the case that the FLE acts as a sludge in these cases, then the need for caution is imperative.

10 See Wilkinson (2013) for arguments to the effect that nudges easily fall into the category of manipulation. In his treatment of influencing how others think and behave with respect to nudges more generally, Sunstein (2016) claims that “an effort to influence people’s choices is manipulative *to the extent that it does not sufficiently engage or appeal to their capacity for reflection and deliberation”* (2016, p. 82, italics in original). He writes that a “manipulator is leading the chooser *to make a choice without sufficiently assessing, on the chooser’s own terms, its costs and benefits*” and that objections to manipulative nudges “reflect a sense that people are not being treated respectfully. Their own capacities and agency – to assess, to weigh, to judge – are not being given appropriate deference” (p. 84, italics in original). If the nudgers are aware of the FLE, and instigate it in others so that others act in line with what the nudger views as the greater good, it is a serious question as to whether the nudger is insufficiently engaging with the person who is being nudged on that person’s own terms.
